# A Leptin Receptor Mutation Which Impairs Fertility in Ewes Causes Delayed Puberty in Male and Female Mice

**DOI:** 10.1210/endocr/bqaf058

**Published:** 2025-03-25

**Authors:** Rebecca A Lord, Megan A Inglis, Jennifer L Juengel, Greg M Anderson

**Affiliations:** Centre for Neuroendocrinology, and Department of Anatomy, University of Otago School of Biomedical Sciences, Dunedin 9016, New Zealand; Centre for Neuroendocrinology, and Department of Anatomy, University of Otago School of Biomedical Sciences, Dunedin 9016, New Zealand; Agricultural Systems and Reproduction, AgResearch Ltd, Invermay Agricultural Centre, Mosgiel 9092, New Zealand; Centre for Neuroendocrinology, and Department of Anatomy, University of Otago School of Biomedical Sciences, Dunedin 9016, New Zealand

**Keywords:** leptin receptor, puberty, reproduction, obesity

## Abstract

Reproductive function is tightly linked to nutritional status due to its high energetic demands. Leptin, a key adipose tissue-derived hormone signalling energy reserves to the brain, integrates metabolic status with the hypothalamic-pituitary-gonadal axis to ensure reproductive function is maintained or suppressed appropriately. Mutations in leptin or its receptor (LepR) are known to cause infertility and obesity in mice. In Davisdale ewes, 2 naturally occurring LepR mutations (R62C and P1019S) were associated with delayed puberty and subfertility, but their effects in males or in other species remain to be determined. This study examined the impact of analogous LepR mutations (A63C and P1018S) in mice using CRISPR-Cas9 gene editing. Puberty onset, adult fertility, and metabolic phenotypes were assessed in wild-type, heterozygous, and homozygous mutant mice. The A63C mutation, located in the extracellular domain of the receptor, resulted in increased body weight and adiposity in females, along with delays in puberty onset in both sexes. Despite these delays, adult reproductive function was maintained. Immunohistochemical analysis revealed no detectable reductions in leptin-induced pSTAT3, pERK1/2, or pmTOR signalling in the hypothalamic arcuate nucleus in either mutant line, indicating these pathways remain largely intact. These findings demonstrate the conserved importance of this region of the leptin receptor for puberty onset and adiposity across species, but also the resilience of leptin signalling in preserving reproductive function despite genetic variation.

The onset of puberty represents a critical milestone in achieving reproductive maturity, characterized by significant hormonal shifts, rapid growth, and the development of reproductive capacity (1, 2). This complex process depends on the intricate interplay of genetic, hormonal, and neurological pathways, with energy availability being a key regulator (2-4). Leptin, a hormone produced by adipose tissue, plays a critical role in linking energy reserves to reproductive development (5-10). Its circulating levels are proportional to total body fat, and it is hypothesized that leptin acts as a signal of energy sufficiency, triggering puberty when a critical threshold is reached (11-13). By interacting with the hypothalamic-pituitary-gonadal (HPG) axis, leptin ensures that reproductive development commences only when sufficient energy reserves are available (14). Disruptions in leptin signalling, resulting from mutations in leptin or its receptor (LepR), have been linked to infertility, severe obesity, and delayed or absent pubertal development in both humans and animal models (12, 15, 16). In 1998, Clément et al reported the first human cases of biallelic LepR mutations, which were associated with early-onset obesity, impaired pubertal progression, and deficiencies in GH and thyrotropin secretion (17). Since this discovery, numerous LepR mutations have been identified in humans, providing critical insights into leptin's role in regulating metabolic and reproductive processes (18-21). Comparable phenotypes have been observed in animal models, such as mice, where LepR mutations similarly disrupt metabolic homeostasis and reproductive function (22-24). These findings highlight leptin's importance in synchronizing metabolic and reproductive processes.

Recent studies have provided further insights into LepR function and its implications for reproductive biology through the identification of novel mutations in sheep. Juengel et al (25). and Haldar et al (26). characterized 2 naturally occurring LepR mutations in the Davisdale sheep breed, each resulting in a single amino acid substitution. Using a candidate gene approach, 2 single nucleotide polymorphisms (SNPs) were identified in the exons of the LepR gene. SNP A, located at position chr1:40787726, induces an arginine-to-cysteine substitution at amino acid 62 (R62C), whereas SNP B, at position chr1:40857869, results in a proline-to-serine substitution at amino acid 1019 (P1019S) (25, 26). Notably, the R62C mutation, situated in the extracellular region of LepR, was predicted to alter receptor structure and potentially impair binding activity (25, 26). Functional implications of these mutations were evident, as ewes homozygous for either mutation demonstrated delayed puberty, reduced ovulation rates, and an inability to conceive during the first breeding cycle, leading to significantly fewer pregnancies (25, 26). The phenotypic effects were more pronounced in individuals carrying the R62C mutation, highlighting its greater impact on reproductive outcomes (25, 26). The R62C and P1019S mutations located in the extracellular and intracellular portion of the LepR were commonly, but not exclusively inherited together, which makes it difficult to ascertain their respective contributions to the reproductive phenotype (25, 26).

These findings underscore the significant impact of LepR mutations on reproductive traits in Davisdale sheep, but their broader implications for other mammalian species remain unclear. Furthermore, the absence of data on males highlights a critical gap in understanding whether these mutations have sex-specific effects. Understanding whether these mutations similarly disrupt puberty onset and fertility in other mammals is crucial for uncovering the conservation of leptin signalling mechanisms across species. To investigate this, CRISPR-Cas gene editing was used to develop engineered mouse models with equivalent A63C and P1018S mutations in the LepR, corresponding to the ovine R62C and P1019S substitutions. These models serve as a powerful tool for investigating the broader biological effects of these novel mutations, shedding light on the complex role of leptin signalling in mammalian reproductive biology.

## Methods

### Animal Care and Ethical Approval

The University of Otago Animal Ethics Committee approved all animal experimental protocols. Mice were group-housed (4 or fewer per cage) in individually ventilated cages within the Biomedical Research Facility at the University of Otago. The environmental conditions were maintained at a controlled temperature, 21 ± 1 °C, and on a 12-hour light/12-hour dark cycle (lights on between 0600 and 1800 h). Mice had ab libitum access to food and water for the duration of the experiment. Mouse lines used were on a C57BL/6J background strain.

### Generation and Genotyping of A63C and P1018S LepR Mutant Mice

A BLAST alignment was used to map the Davisdale sheep R62C and P1019S LepR mutations relative to the mouse genome. It was determined that the murine A63C and P1018S LepR mutations would result in an identical amino acid substitution as that seen in Davisdale sheep (25, 26). To establish a mouse model, point mutations (A63C or P1018S) were introduced into the mouse LepR locus using CRISPR/Cas-mediated genome editing by Cyagen (Santa Clara, CA, USA). The mouse LepR gene is located on chromosome 4 and comprises 19 exons (GenBank accession number: NM_146146.2). The A63C mutation is located in exon 3, where a guide RNA (gRNA) targeting vector and donor oligo, flanked by 120 base pair (bp) homologous sequences, were designed for precise editing. The A63C substitution (GCT to TGT) was incorporated via homology-directed repair, accompanied by synonymous mutations (A57: GCC to GCG, L60: TTG to CTC) to prevent sequence recleavage postediting. Similarly, the P1018S mutation, located in exon 19, was targeted using a gRNA vector and donor oligo with flanking homologous sequences, introducing the P1018S substitution (CCA to TCA). Synonymous mutations (S1013: TCC to TCG, R1020: AGG to AGA) were introduced to prevent gRNA rebinding. Cas9, gRNA, and donor oligo co-injection into fertilized eggs facilitated the generation of A63C and P1018S LepR knock-in mice. Heterozygous breeders were used to generate mice homozygous (+/+), heterozygous (+/−) or wild-type (WT) for each LepR mutation, respectively. Genotyping of pups was performed using PCR followed by sequencing to confirm successful targeting.

The A63C mutant mouse line was identified using the following PCR primers and annealing temperature: 5′ ACCGAACACAACCGATGACT 3′ (mutant forward primer), 5′ ACTCAGGAACGTAGATACCACT 3′ (mutant reverse primer), annealing temperature 55 °C; size of uncut product 128 bp. The P1018S mutant mouse line was identified using the following PCR primers and annealing temperature: 5′ GTCCTGTCAGCAACTGCATCT 3′ (mutant forward primer), 5′ GGAAAAATGTCTGGGCCTCTG 3′ (mutant reverse primer), annealing temperature 55 °C; size of uncut product 93 bp. Both samples were run under the following conditions: 95 °C (3 minutes), [95 °C (30 seconds), 55 °C (1 minute), 72 °C (45 seconds)] × 35. 72 °C (5 minutes), cool (10 °C). Following PCR, MnII (A63C) or XhoI (P1018S) restriction enzymes were used to differentiate between cut and uncut PCR products. While MnII cuts the wild-type allele, XhoI specifically targets and cuts the mutant allele.

Because of limited WT breeders at the project's onset, WT A63C and P1018S mice were pooled into single male and female groups to increase sample sizes and improve statistical power. As both lines were derived from the same C57BL/6J background strain within 3 to 5 generations, pooling was unlikely to introduce significant variability.

### Measuring Metabolic and Reproductive Parameters in A63C and P1018S LepR Mutant Mice

Mice were weighed weekly from 3 to 8 weeks of age, with final body weight recorded on the day of perfusion. Abdominal fat, comprising mesenteric, retroperitoneal, and gonadal fat stores were dissected and measured for adiposity evaluation at the end of the experiment.

Puberty onset was assessed in all mice from weaning at 20 days of age. In females, genitalia were monitored daily for vaginal opening, an external marker of puberty initiation. Once observed, saline lavages were used to collect daily vaginal cytology samples until the first estrous smear, characterized by clusters of nonnucleated, cornified epithelial cells, indicating puberty completion. In males, genitalia were checked daily for preputial separation, a marker of puberty onset, determined by applying gentle pressure to observe separation of the penis from the prepuce.

Estrous cyclicity in adult females was assessed through daily vaginal cytology from 6 to 8 weeks of age, over a 10-day period. The average cycle length was calculated by determining the interval between 2 estrous phases for each mouse and averaging it over the data collection period. At the end of the experiment, reproductive organs were collected and weighed in both sexes, including the uterus and ovaries in females and the testes and seminal vesicles in males.

### Assessment of pSTAT3, pmTOR and pERK1/2 Signalling Using Chromogen Immunohistochemistry

To assess the impact of LepR mutations on cell signalling responses to a leptin bolus, a subset of male mice were fasted overnight for 12 hours to reduce endogenous leptin levels. They were then challenged with a submaximal leptin dose (0.02 mg/kg, subcutaneously) 1 hour before perfusion, allowing for subtle variations in LepR signalling to be detected through chromogen immunohistochemistry. Ten minutes before perfusion, sodium pentobarbital (100 mg/kg, IP) was administered as an anesthetic overdose. Mice were perfused transcardially with 4% paraformaldehyde in 0.1 M PBS (pH 7.4). The brains were then collected, postfixed in paraformaldehyde, and transferred to a 30% sucrose solution. Coronal sections were cut at 30 μm in series of 3.

Immunohistochemistry was performed on free-floating arcuate brain sections to assess leptin-induced phosphorylated signal transducer and activator of transcription 3 (pSTAT3), phosphorylated extracellular signal-related kinase 1/2 (pERK1/2), and phosphorylated mammalian target of rapamycin (pmTOR). Following antigen retrieval at 90 °C for 15 minutes in 1 mM EDTA (pH 8.0) for STAT3 or 10 minutes in 0.01 M Tris-HCl (pH 10.0) for mTOR and ERK1/2, tissues were incubated with primary antibodies at 4 °C for 48 hours: pSTAT3 (anti-pSTAT3 [Tyr705] 1:5000, Cell Signalling, RRID: AB_2491009), pERK1/2 (anti phospho-p44/42 MAPK [Thr202/Tyr204] 1:2000, Cell Signalling, RRID: AB_331646), and anti-pmTOR (pmTOR [Ser2448] 1:2000, Cell Signalling, RRID: AB_490932). We validated these antibodies in previous studies using brain tissue from knockout mice; this resulted in complete absence of staining. Tissues were then incubated in a biotinylated secondary antibody for 1 hour (goat anti-rabbit IgG, 1:1000; Vector Laboratories, RRID:AB_2313606), followed by Vector Elite avidin-biotin complex solution for 30 minutes (Vector Laboratories) then 3,3′-diaminobenzidine solution (1 urea $H_{2}O_{2}$ and 1 SIGMA*FAST* 3,3′-diaminobenzidine tablet dissolved in 20 mL of distilled $H_{2}O$, Sigma-Aldrich) that was nickel-enhanced (160 mg $\mathrm{Ni}(NH_{4}{)}_{2}SO_{4}$) to visualize immunohistochemical black staining. Tissues were then washed and mounted onto gelatin-coated microscope slides and left to dry overnight before being coverslipped with DPX mounting medium. Sections were imaged using a light microscope (Olympus BX51), and immunoreactive cells in the arcuate nucleus were counted bilaterally with ImageJ FIJI software. For each mouse, cell counts from 3 to 4 sections were averaged to obtain a single value.

### Statistical Analyses

Statistical analyses were performed, and data graphed using Prism software 9.0 (GraphPad). All data are graphed and presented as mean ± SEM. Discrete data with a limited range (such as the date of puberty onset) cannot be assumed to follow a normal distribution. As a result, they were subjected to analysis using the nonparametric Kruskal-Wallis test. For all other data, a Student *t*-test was used to compare 2 groups and a 1-way ANOVA with Tukey multiple comparisons post hoc analysis was used to compare 3 groups. A *P* value <.05 was considered statistically significant.

## Results

### A63C LepR Mutation Causes Increased Body Weight and Adiposity in Females and Delays Puberty in Both Sexes

**Figure 1.**
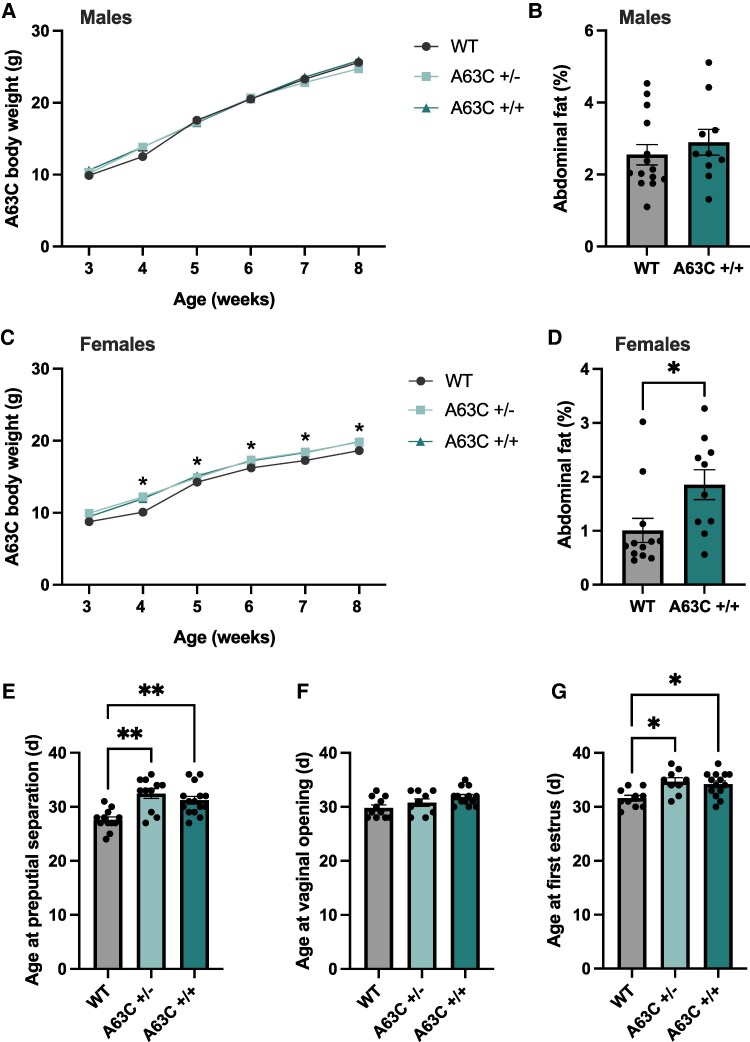
A63C LepR mutation causes increased body weight and adiposity in females and delayed puberty onset in male and female mice. Body weight trajectories from weaning to 8 weeks in male (A) and female (C) wildtype, A63C+/− and A63C+/+ mice. Percentage of abdominal adiposity measured in A63C+/+ and wildtype male (B) and female (D) mice. Average age at preputial separation (E) in males in conjunction with vaginal opening (F) and first estrus (G) in females. Wildtype: n = 12-14, A63C+/−: n = 9-11, A63C+/+: n = 13-14 per group. Data presented as mean ± SEM. Bodyweight analyzed using a 1-way ANOVA with Tukey post hoc test, *P < .05. Adiposity analyzed using a Student t test, *P < .05. Puberty onset was analyzed using a nonparametric Kruskal-Wallis test, *P < .05, **P < .01. Abbreviations: A63C+/+, homozygous; A63C+/−, heterozygous; WT, wildtype.

Body weight was monitored in both sexes from weaning to assess the effect of the A63C LepR mutation on metabolic regulation. In males, there was a significant main effect of time (F_5,7_ = 291.9, *P* < .001) on body weight, but no main effect of mutation or time × mutation interaction. Tukey post hoc test for multiple comparisons revealed no difference in body weight in A63C+/− and A63C+/+ males relative to the controls (Fig. 1A, *P* > .05). In females, there was a significant main effect of time (F_5,71_ = 292, *P* < .001) and mutation (F_2,93_ = 25.8, *P* < .001) on body weight, but no time × mutation interaction. Tukey post hoc test for multiple comparisons revealed A63C+/− and A63C+/+ females had significantly increased body weight than their WT counterparts from 4 weeks, respectively (Fig. 1C, *P* < .01). Additionally, the effect of the A63C LepR mutation on adiposity was measured following euthanasia. In males, there was no difference in the percentage of adiposity between A63C+/+ and WT mice (Fig. 1B). In females, A63C+/+ mice showed a significant elevation in adiposity over their WT littermates, in support of the bodyweight phenotype observed (Fig. 1D, *P* = .026).

Puberty onset was measured from weaning (PND20) in males and females. A63C+/− males showed a 4.8 days delay (*P* = .0011) and A63C+/+ males showed a 3.6-day delay (*P* = .008) in preputial separation compared to WT mice (Fig. 1E). In females, the timing of vaginal opening which is considered an early marker of puberty initiation (27), did not differ between groups (Fig. 1F, *P* = .06). However, the onset of the first estrus, considered a marker of puberty completion indicating the first ovulation (28), was significantly delayed by 3.1 days in A63C+/− and 2.6 days in A63C+/+ females compared to controls (*P* = .017 and *P* = .024 respectively; Fig. 1G).

### A63C LepR Mutation has no Effect on Estrous Cyclicity or Reproductive Organ Weights

**Figure 2.**
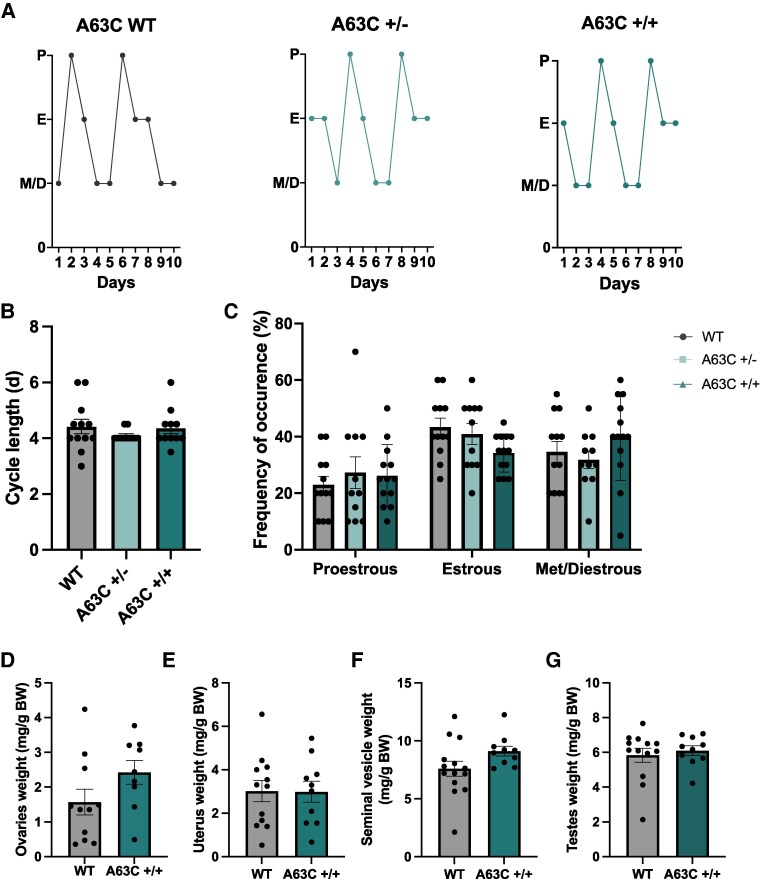
A63C LepR mutation does not affect estrous cyclicity in females or reproductive organ weights in male and female mice. Representative estrous cycle profiles for wildtype, A63C+/−, and A63C+/+ mice (A). Quantitative analysis of estrous cycle length (B) and percentage of time spent in each cycle stage (C) among genotypes. Weight of the ovaries (D) and uterus (E) in wildtype and A63C+/+ females as a proportion of final body weight. Weight of the combined seminal vesicles (F) and testes (G) in A63C+/+ and wildtype males as a proportion of final body weight. Wildtype: n = 12-14, A63C+/−: n = 11, A63C+/+: n = 13-14 per group. Data presented as mean ± SEM. Cycle length (B) was analyzed using a nonparametric Kruskal-Wallis test. Cycle stage (C) was analyzed using a 1-way ANOVA with Tukey post hoc test. Reproductive organ weights (D–G) analyzed using a Student t test. Abbreviations: A63C+/+, homozygous; A63C+/−, heterozygous; BW, body weight; D, diestrus; E, estrus; M, metestrus; *P*, proestrus; WT, wildtype.

Reproductive physiology in females was assessed through vaginal cytology to examine estrous cyclity. As evidenced by the representative examples shown, WT, A63C+/−, and A63C+/+ females all showed normal estrous cyclicity (Fig. 2A). No significant difference in cycle length was observed (Fig. 2B), characterized by the interval between 2 successive estrous smears. Additionally, there was no difference in the percentage of time spent in each estrous cycle stage between groups (Fig. 2C).

Reproductive organs were dissected and weighted following euthanasia to provide an indication of sex steroid hormone synthesis and gonadal development in WT and A63C+/+ mice. Uterus weight (used as a proxy for circulating estradiol) and combined ovarian weights did not differ between WT and A63C+/+ females (Fig. 2D–2E; *P* = .9 and *P* = .1, respectively). Additionally, combined seminal vesicle (used as a proxy for circulating testosterone) and testes weights were comparable between WT and A63C+/+ males (Fig. 2F–2G; *P* = .09 and *P* = .6, respectively).

### P1018S LepR Mutation has Modest Effects on Body Weight in Both Sexes, and Males Heterozygous for the Mutation Have a Significant Delay in Puberty Onset

**Figure 3.**
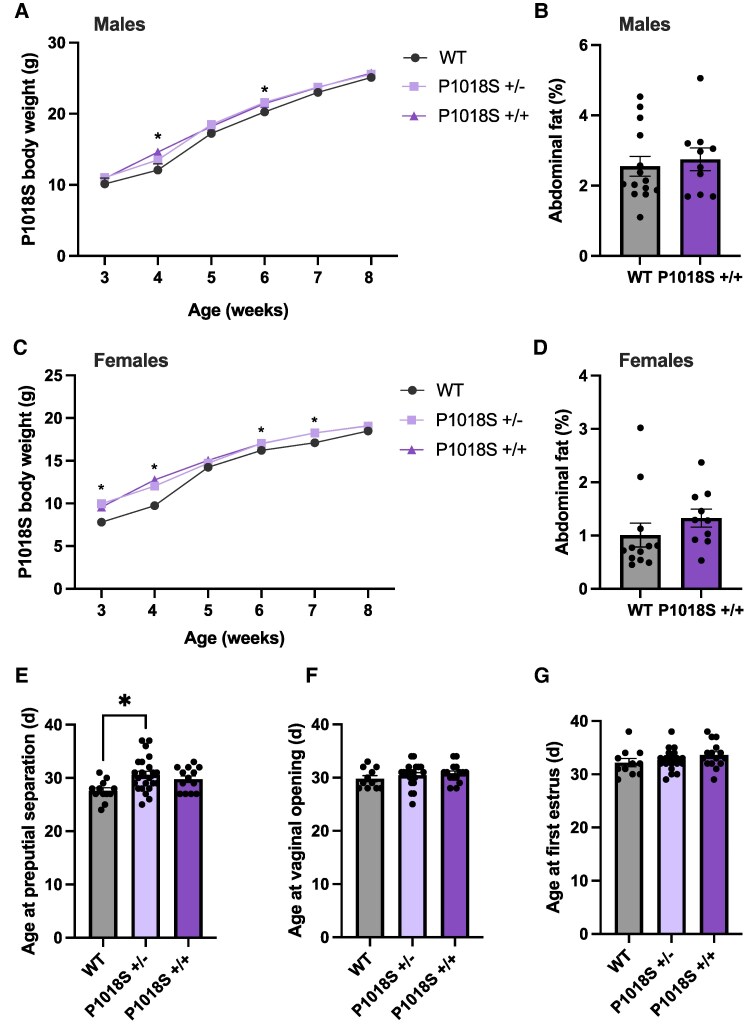
P1018S LepR mutation modestly affects body weight and delays preputial separation in heterozygous males. Body weight trajectories from weaning to 8 weeks in male (A) and female (C) wildtype, P1018S+/−, and P1018S+/+ mice. Percentage of abdominal adiposity measured in P1018S+/+ and wildtype males (B) and females (D) mice. Average age at preputial separation (E) in males in conjunction with vaginal opening (F) and first estrus (G) in females. Wildtype: n = 11-12, P1018S+/−: n = 19-22, P1018S+/+: n = 13-15 per group. Data presented as mean ± SEM. Bodyweight analyzed using a 1-way ANOVA with Tukey post hoc test, *P < .05. Adiposity analyzed using a Student t test. Puberty onset was analyzed using a nonparametric Kruskal-Wallis test, *P < .05. Abbreviations: P1018S+/+, homozygous; P1018S+/−, heterozygous; WT, wildtype.

Similarly, body weight was monitored in both males and females from the time of weaning (PND20) to evaluate the impact of the P1018S LepR mutation on metabolic regulation. In males, there was a significant main effect of time (F_5,217_ = 393.6, *P* < .001) and mutation (F_2,217_ = 8.9, *P* < .001), but no time × mutation interaction. Tukey post hoc test for multiple comparisons revealed that P1018S+/+ males had significantly increased body weight at 4 weeks (Fig. 3A, *P* = .005) and P1018S+/− at 6 weeks (Fig. 3A, *P* = .03) compared to WT males, but otherwise had comparable body weights at all other time points. In females, there was a significant main effect of time (F_5,219_ = 371.4, *P* < .001) and mutation (F_2,219_ = 31.3, *P* < .001) on bodyweight. As a result of this, there was also a time × mutation interaction (F_10,219_ = 2.3, *P* = .16). Tukey post hoc test for multiple comparisons revealed that P1018S+/− and P1018S+/+ females had significantly elevated body weight between 3 and 4 weeks of age and again at 6 to 7 weeks of age compared to aged matched controls (*P* < .001 and *P* < .05, respectively; Fig. 3C). At all other timepoints, bodyweight was comparable between groups.

The influence of the P1018S LepR mutation on adiposity was also assessed after euthanasia. In males, there was no difference in the percentage of adiposity between P1018S+/+ and WT mice (Fig. 3B, *P* = .6). Additionally, there was no observable difference in adiposity between P1018S+/+ females and their WT counterparts (Fig. 3D; *P* = .3).

Puberty onset was assessed in males and females beginning at weaning (PND20). P1018S+/− males showed a 3-day delay (Fig. 3E; *P* = .012) in preputial separation compared to WT mice. There was no difference in age at preputial separation between P1018S+/+ and WT males. Additionally, P1018S+/− and P1018S+/+ females showed no difference in the age at vaginal opening, or first estrus compared to WT mice (Fig. 3F and 3G, respectively).

### P1018S LepR Mutation has no Effect on Estrous Cyclicity or Reproductive Organ Weights

**Figure 4.**
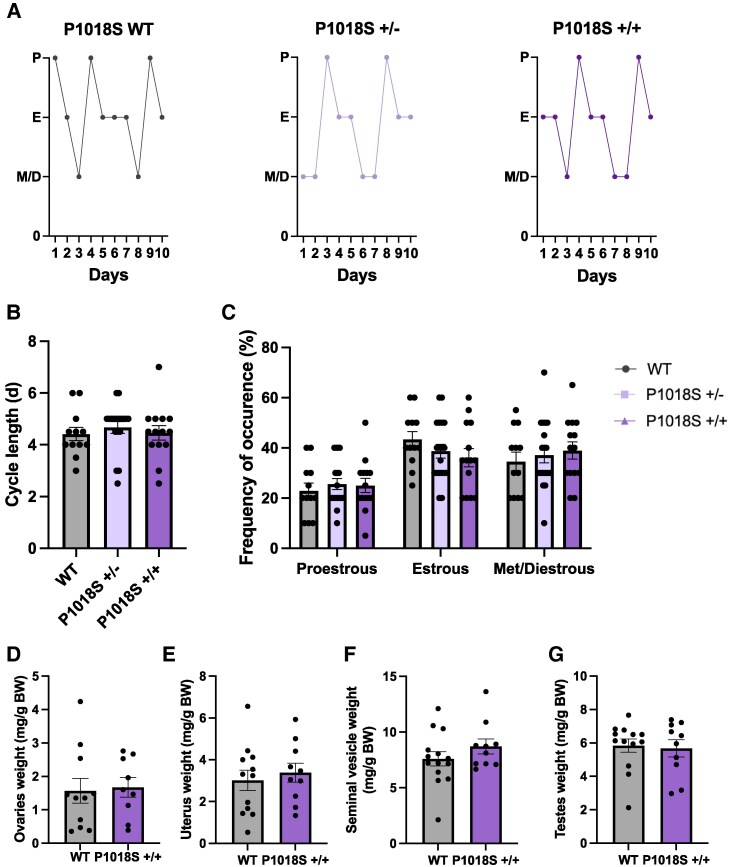
P1018S LepR mutation does not alter estrous cyclicity in female mice or reproductive organ weights in either sex. Representative estrous cycle profiles for wildtype, P1018S+/−, and P1018S+/+ female mice (A). Quantitative analysis of estrous cycle length (B) and percentage of time spent in each cycle stage (C) among genotypes. Weight of the ovaries (D) and uterus (E) in wildtype and P1018S+/+ females as a proportion of final body weight. Weight of the combined seminal vesicles (F) and testes (G) in P1018S+/+ and wildtype males as a proportion of final body weight. Wildtype: n = 11-12, P1018S+/−: n = 19, P1018S+/+: n = 13-14 per group. Data presented as mean ± SEM. Cycle length (B) was analyzed using a nonparametric Kruskal-Wallis test. Cycle stage (C) was analyzed using a 1-way ANOVA with Tukey post hoc test. Reproductive organ weights (D-G) analyzed using a Student *t*-test. Abbreviations: BW, body weight; D, diestrus; E, estrus; M, metestrus; *P*, proestrus; P1018S+/+: homozygous; P1018S +/−: heterozygous; WT, wildtype.

Female reproductive physiology was evaluated using vaginal cytology to investigate estrous cycling. As illustrated by the representative examples, WT, P1018S+/−, and P1018S+/+ females all exhibited normal estrous cycles (Fig. 4A). No notable differences in cycle length were detected (Fig. 4B), defined by the time between 2 consecutive estrous smears. Similarly, there was no variation in the percentage of time spent in each stage of the estrous cycle across the groups (Fig. 4C).

Following euthanasia, reproductive organs were dissected and weighed to assess sex steroid hormone production and gonadal development in WT and P1018S+/+ mice. Uterine weight (a marker for circulating estradiol) and combined ovarian weights showed no significant differences between WT and P1018S+/+ females (Fig. 4D–4E; *P* = .6 and *P* = .8, respectively). Similarly, combined seminal vesicle weights (a marker for circulating testosterone) and testes weights were comparable between WT and P1018S+/+ males (Fig. 4F–4G; *P* = .3 and *P* = .8, respectively).

### Assessing LepR Signalling Molecules in A63C and P1018S LepR Mutant Mice

Immunohistochemical analysis was conducted to evaluate leptin-induced pSTAT3, pERK1/2, and pmTOR signalling in A63C and P1018S mutant mice (+/+) relative to WT controls.

**Figure 5.**
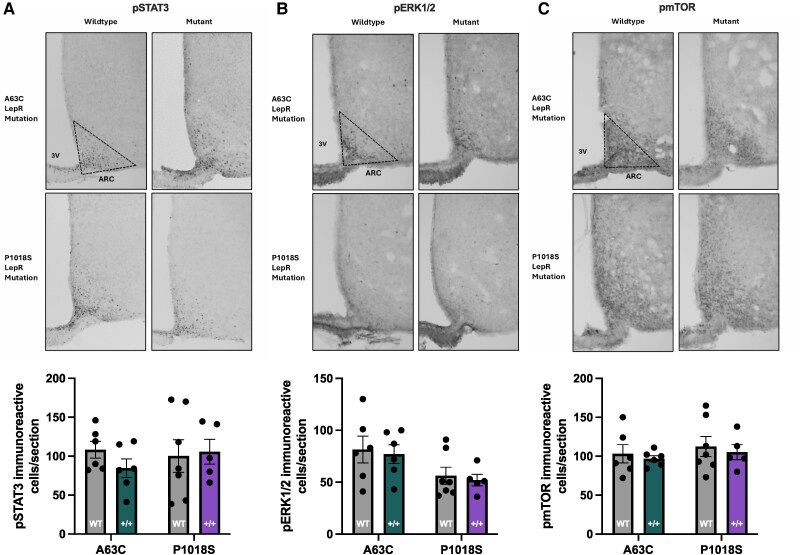
Quantification of immunoreactive pSTAT3, pERK1/2 and pmTOR cells in the arcuate nucleus in response to A63C and P1018S LepR mutation. Leptin-induced pSTAT3, pERK1/2, and pmTOR was quantified in the arcuate nucleus (ARC) in response to a bolus of leptin (0.02 mg/kg) following an overnight fast. Representative images of pSTAT3 (A) pERK1/2 (B) and pmTOR (C) staining within the ARC are shown for A63C and P1018S wildtype and mutant mice. Quantification of pSTAT3 (A), pERK1/2 (B), and pmTOR (C) immunoreactive cells within the ARC counted manually using ImageJ is also shown. A63C: n = 6 per group, P1018S: n = 5-7 per group. Data were analyzed using a 1-way ANOVA followed by Tukey post hoc test. Data presented as mean ± SEM. pERK1/2, phosphorylated extracellular signal-related kinase 1/2; pSTAT3, phosphorylated signal transducer and activator of transcription 3; pmTOR, phosphorylated mammalian target of rapamycin; 3V: third ventricle. The triangle indicates the area containing the ARC used for counting pSTAT3, pERK1/2, or pmTOR staining. Scale bar = 100 µm.

When assessing pSTAT3, there was no significant main effect of genotype. No significant differences were observed in the number of pSTAT3 immunoreactive cells for either mutation. In both A63C and P1018S mice, WT and mutant pSTAT3 counts were comparable (Fig. 5A; *P* = .31 and *P* = .81, respectively). No significant difference was observed between A63C and P1018S mutants (Fig. 5A; *P* = .39). Similarly, pERK1/2 staining revealed no significant differences between WT and mutant mice for either LepR mutation and there was no significant main effect of genotype. Both A63C and P1018S mutants had similar pERK1/2 staining to their WT counterparts (Fig. 5B, *P* = .74 and *P* = .76; respectively). While P1018S mutants exhibited ∼25% fewer pERK1/2 cells than A63C mutants (F_1,20_ = 6.8, *P* < .05), this was not significant in post hoc comparisons (Fig. 5B; *P* = .093). Finally, pmTOR staining also showed no significant main effect of genotype. In A63C and P108S mice, WT and mutant counts were comparable (Fig. 5C; *P* = .68 and *P* = .63, respectfully). No differences were observed between A63C and P1018S mutants (Fig. 5C, *P* = .60). These results indicate that leptin-induced signalling through pSTAT3, pERK1/2, and pmTOR is not significantly disrupted in either mutant line.

## Discussion

This research aimed to further characterize 2 naturally occurring LepR mutations, R62C and P1019S, identified in a line of Davisdale sheep, and to investigate whether these mutations, known to cause subfertility in sheep, were associated with defects in leptin signalling, puberty onset, and adult fertility in engineered mouse lines carrying the equivalent A63C and P1018S LepR mutations (25, 26). These findings reveal that the A63C LepR mutation, in particular, is associated with delayed puberty onset observed in both male and female mice, and mild obesity in females.

Leptin regulates feeding and energy expenditure, with disruptions in leptin receptor signalling often leading to obesity (29, 30). Although Davisdale ewes carrying these SNPs exhibited increased body weight, A63C mutant males showed no significant weight gain compared to wildtype controls (25, 26). In contrast, P1018S LepR mutant males displayed a modest increase in body weight at 4 and 6 weeks of age. Notably, A63C and P1018S mutant females (+/+ and +/−) demonstrated significant weight increases, with A63C females showing elevated body weight from 4 weeks of age and P1018S females exhibiting increases during 3 to 4 and 6 to 7 weeks of age. Abdominal fat mass, assessed in WT and homozygous (+/+) A63C and P1018S mice, provided further insights not available from the Davisdale sheep studies (25, 26). Female mice homozygous for the A63C mutation displayed significantly increased abdominal fat compared to WT controls. No such differences were observed in male mice, where abdominal fat levels were comparable across genotypes. These findings suggest that the A63C LepR mutation influences adiposity in a sex-specific manner. This effect may stem from partial disruption of the HPG axis, potentially impairing production of estradiol, a hormone known to have a strong inhibitory effect on abdominal adiposity protecting female mice from obesity (31, 32). However, the lack of difference in uterine mass does not support this. Instead, the increased adiposity in females is more likely to be due to increased food intake and/or reduced energy expenditure, as these are also highly responsive to leptin signalling (7, 33, 34). Unfortunately, we were not able to conduct measurements to assess these functions in the current study. It would be intriguing to explore whether a more pronounced metabolic phenotype could arise from subjecting animals to a high-fat diet, potentially inducing heightened leptin signalling and subsequent leptin resistance (35). Leptin receptor mutations may further exacerbate or accelerate the onset of leptin resistance in this situation. This would be worth investigating in future studies.

Puberty onset was assessed to determine whether these LepR mutations in mice lead to delays seen in Davidsdale ewes (25, 26). A63C+/+ or +/− males, and P1018S+/− males, exhibited a significant delay in preputial separation compared to WT controls, consistent with previous findings in LepR-null models (22). These data were unique to this study because this had not been previously assessed in the sheep. This androgen-dependent process likely reflects a delay in activation of the HPG axis, leading to reduced testosterone production (36). Interestingly, heterozygous males showed a more pronounced delay than homozygous males, further emphasizing the functional significance of this mutation. Larger sample sizes would be required to more thoroughly investigate this apparent effect of zygosity. In females, the A63C LepR mutation caused a significant delay in first estrus but not in vaginal opening, while the P1018S mutation had no effect. Previous studies using LepR-null mice have shown that first estrus, unlike vaginal opening, is leptin-dependent (22). Since first estrus signifies the occurrence of ovulation and the attainment of sexual maturity, it is plausible that leptin's permissive role in puberty is more critical for the completion of puberty (first estrus) rather than its initiation (vaginal opening) (12, 37-41). The observed delays in both preputial separation and first estrus in A63C+/+ and +/− mice support the hypothesis that this specific mutation likely underpins the reproductive phenotype in Davisdale sheep (25, 26). Notably, these delays occurred independently of body weight changes, suggesting that disrupted puberty onset can be influenced by impaired leptin signalling independently of leptin's metabolic effects.

The estrous cycle, driven by fluctuations in ovarian progesterone and estrogen, is a key indicator of reproductive function in female mice (42). These findings revealed no significant differences in cycle length, stage frequency, or overall cycling patterns over a 10-day period among A63C and P1018S LepR-mutant females (+/+ or +/−) compared to WT controls. All groups exhibited normal estrous cycling. These results suggest that neither the A63C nor P1018S LepR mutations impact regular estrous cyclicity. Comparable data from Davisdale ewes are unavailable and would be complicated by their seasonal breeding patterns, unlike the nonseasonal, continuously cycling nature of female mice. Additionally, reproductive organ weights were measured in homozygous and WT A63C and P1018S LepR mice to assess gonadal development and infer sex steroid levels. In males, no significant differences in testicular or seminal vesicle weights were observed between A63C or P1018S homozygous and WT groups. Similarly, in females, ovarian and uterine weights did not differ between homozygous A63C or P1018S mutants and WTs, indicating normal GnRH-mediated gonadotropin release and estradiol or androgen production. This lack of effect is likely due to redundant regulatory pathways maintaining GnRH stimulation and gonadotropin release (43, 44). In Davisdale sheep, subfertility in homozygous ewes was attributed to reduced ovulation rates but did not include direct measures of uterine or ovarian weights (25, 26). While subfertility was evident in Davisdale ewes with reduced ovulation and conception rates linked to these LepR mutations, preliminary breeding data in these mice (not shown) showed no clear impact on litter size or number of litters produced. However, the small sample size limits definitive conclusions, and a dedicated fecundity study is needed to validate these findings.

To investigate the leptin signalling pathways potentially mediating the delayed puberty phenotype observed in mice, brain tissue was analyzed for leptin signalling molecules pSTAT3, pERK1/2, and pmTOR at 60 minutes following an exogenous leptin challenge. STAT3 activation in the arcuate nucleus is known to be maximal between 30 to 60 minutes after leptin treatment (45). The results indicate that the A63C and P1018S LepR mutations do not significantly downregulate STAT3, ERK1/2, or mTOR signalling, despite the A63C mutation being associated with a delayed puberty phenotype. The implication of this is that the effects of these LepR mutations on downstream signalling is relatively mild in mice. The measurements of LepR signaling were conducted in males, whereas the most obvious puberty and metabolic effects of the mutations were seen in females. Therefore, it remains possible that disrupted leptin signaling occurs in LepR-mutant female mice. Furthermore, a single amino acid substitution may not entirely disrupt the function of these signalling molecules but could reduce their efficiency or stability. Although leptin binding and downstream signalling might be impaired, the receptor likely retains enough functionality to maintain essential roles, albeit less effectively. As a result, physiological processes regulated by these pathways, such as energy balance, body weight, and reproductive functions, may remain largely intact. This moderate impairment may not be sufficient to produce detectable changes in the immunoreactive staining of these signalling molecules. However, it remains possible that other less-studied leptin signalling pathways, such as CREB-regulated transcription coactivator 1 (46-48), are impaired by these mutations. This remains an active area of research for our group. In addition to confirming the canonical role of STAT3, our unpublished findings suggest a minor role for neuronal CREB-regulated transcription coactivator 1 but not for mTOR or ERK2.

It is also interesting to note that although the A63C mutation, located in the extracellular region of the LepR, would presumably affect both short and long isoform LepR binding and signalling, the P1018S mutation, positioned at the intracellular end of the LepR, would exclusively impact long-form LepR signalling (25, 26). Notably, the P1018S mutation is situated just downstream of the Tyr amino acid residue 1138 (Y1138), which is essential for STAT3 phosphorylation. Although this may explain the absence of significant effects of this mutation on leptin-induced STAT3 phosphorylation in this study, it remains possible that mutations in close proximity to the STAT3 activation site subtly modulate LepR function. In this study, LepR mutations were analyzed individually; however, in sheep studies, where these mutations occur naturally, multiple mutations were typically present, potentially leading to a more pronounced phenotype.

Puberty is a critical process vital for reproductive success, safeguarded by multiple mechanisms that ensure its timely initiation and progression. The A63C and P1018S LepR mutations highlight the system's resilience because they cause minor delays in puberty and mild adiposity while not noticeably affecting adult reproductive function. This suggests that leptin receptor function, though modestly impaired, remains sufficiently robust to sustain reproductive functionality once sexual development has occurred (49). Biological systems often employ compensatory mechanisms to maintain homeostasis and essential functions despite genetic disruptions. When primary signalling pathways are affected, alternative pathways may compensate to ensure continuity of processes like body weight regulation and puberty onset (50-53). This redundancy within signalling networks allows key physiological functions to persist, even in the presence of genetic variation. These findings emphasize the cooperative and adaptive nature of leptin's regulatory role in reproduction, offering insights into the mechanisms underlying metabolic infertility and puberty attainment.

## Data Availability

Some or all datasets generated during and/or analyzed during the current study are not publicly available but are available from the corresponding author on reasonable request

## Abbreviations

bp: base pair
gRNA: guide RNA
HPG: hypothalamic-pituitary-gonadal
LepR: leptin or its receptor
pERK1/2: phosphorylated extracellular signal-related kinase ½
pmTOR: phosphorylated mammalian target of rapamycin
pSTAT3: signal transducer and activator of transcription 3
SNP: single nucleotide polymorphism
WT: wild-type
